# Experiences of Internet-Based Stepped Care in Individuals With Cancer and Concurrent Symptoms of Anxiety and Depression: Qualitative Exploration Conducted Alongside the U-CARE AdultCan Randomized Controlled Trial

**DOI:** 10.2196/16547

**Published:** 2020-03-30

**Authors:** Anna Hauffman, Sven Alfonsson, Helena Igelström, Birgitta Johansson

**Affiliations:** 1 Department of Immunology, Genetics and Pathology Section of Experimental and Clinical Oncology Uppsala University Uppsala Sweden; 2 Department of Women's and Children's Health Section of Clinical Psychology in Healthcare Uppsala University Uppsala Sweden; 3 Centre for Psychiatry Research Department of Clinical Neuroscience Karolinska Institute and Stockholm Health Care Services Stockholm Sweden; 4 Department of Neuroscience Section of Physiotherapy Uppsala University Uppsala Sweden

**Keywords:** internet-based stepped care, internet-based interactive health communication application, internet-based intervention, telemedicine, patient portals, oncology nursing, self care, psychoeducation

## Abstract

**Background:**

Individuals with newly diagnosed cancer may experience impaired health in several aspects and often have a large need for information and support. About 30% will experience symptoms of anxiety and depression, with varying needs of knowledge and support. Despite this, many of these patients lack appropriate support. Internet-based support programs may offer a supplement to standard care services, but must be carefully explored from a user perspective.

**Objective:**

The purpose of this study was to explore the participants’ perceptions of the relevance and benefits of an internet-based stepped care program (iCAN-DO) targeting individuals with cancer and concurrent symptoms of anxiety and depression.

**Methods:**

We performed a qualitative study with an inductive approach, in which we used semistructured questions to interview 15 individuals using iCAN-DO. We analyzed the interviews using content analysis.

**Results:**

The analysis found 17 subcategories regarding the stepped care intervention, resulting in 4 categories. Participants described the need for information as large and looked upon finding information almost as a survival strategy when receiving the cancer diagnosis. iCAN-DO was seen as a useful, reliable source of information and support. It was used as a complement to standard care and as a means to inform next of kin. Increased knowledge was a foundation for continued processing of participants’ own feelings. The optimal time to gain access to iCAN-DO would have been when being informed of the diagnosis. The most common denominator was feeling acknowledged and supported, but with a desire for further adaptation of the system to each individual’s own situation and needs.

**Conclusions:**

Users saw the internet-based stepped care program as safe and reliable and used it as a complement to standard care. Similar interventions may gain from more personalized contents, being integrated into standard care, or using symptom tracking to adjust the contents. Offering this type of program close to diagnosis may provide benefits to users.

**Trial Registration:**

ClincalTrials.gov NCT-01630681; https://clinicaltrials.gov/ct2/show/NCT01630681

## Introduction

### Background

At the time of a cancer diagnosis and along the disease trajectory, individuals often have large information needs and may strive to get a fuller picture of what has befallen them and what awaits them [1]. The period around diagnosis is often described as frightening, with both new knowledge and support being needed to make the situation more predictable and manageable. Common reactions are shock and denial, followed by anger and symptoms of anxiety or depression [2]. Anxiety and depression may be part of the initial reaction and subside with time, but may also be persistent [3-5]. Targeted psychosocial interventions, especially those involving psychoeducation, may be helpful [6], as they aim to normalize, confirm, and explain feelings and symptoms, providing the individual with an explanatory model. Nevertheless, it is becoming increasingly common for people with cancer to be cared for as outpatients [7], thus spending more time at home during the period of illness. This may be perceived as positive, but it also leads to more limited possibilities of support from health professionals. One way of reaching people, regardless of the route of care delivery, is through electronic health (eHealth) interventions, including internet-based interactive health communication applications (IHCAs). An IHCA may be described as a set of components that offer information, support, and behavior change interventions. Further, internet-based IHCAs can provide relevant, quality-assured information and support when the individual feels ready for it or has time to access a computer or mobile device. IHCAs for people with cancer provide a way of offering support that may improve, for example, distress levels, social support, symptoms of fatigue, and health literacy [8,9].

### The Uppsala University Psychosocial Care Program

The Uppsala University Psychosocial Care Program (U-CARE) [10] is a strategic research venture, in the fields of caring science, psychology, and computer science, supported by the Swedish government. Studies within U-CARE concern the psychosocial consequences of somatic diseases and the support that the affected individuals may need.

### The U-CARE Portal

All interventions and the collection of patient-reported data in U-CARE take place via the U-CARE Portal, developed within U-CARE. The U-CARE Portal is an internet-based infrastructure that enables delivery of care such as self-care programs and psychological treatment within clinical studies and is not tethered to personal health records.

### Objective

Using qualitative methods as part of the evaluation of complex interventions is increasingly common in order to gain further knowledge of aspects important to participants. The qualitative exploration may help explain the findings of the trial and aid understanding of the processes needed for change and implementation [11]. Since it can be difficult to predict how the intervention may work, as well as what users need and want, the qualitative exploration is an important measure to ensure that the intervention serves its purpose [12,13]. The aim of this study was to explore the participants’ perceptions of the relevance and benefits of iCAN-DO.

## Methods

### U-CARE AdultCan

We recruited the informants in this study from AdultCan [14], a randomized controlled trial (RCT) (NCT-01630681) within U-CARE, targeting individuals with newly diagnosed breast cancer, colorectal cancer, or prostate cancer and with symptoms of anxiety or depression, or both. AdultCan aimed to investigate the effects of an internet-based IHCA (iCAN-DO), including a stepped care intervention for symptoms of anxiety and depression. iCAN-DO was developed in collaboration between the research group, staff in clinical cancer care, and individuals with lived experience of cancer. The main purpose of this collaboration, described in detail in a previous article [15], was to target iCAN-DO to the needs of individuals with cancer and concurrent symptoms of anxiety and depression. iCAN-DO is 1 intervention comprising 2 steps (Table 1): interactive support (step 1) based on psychoeducation and assumptions from Orem’s self-care deficit nursing theory [16], social cognitive theory, and additional internet-delivered cognitive behavioral therapy (iCBT, step 2) for individuals with persistent symptoms of anxiety and depression despite the support provided in step 1. Individuals with newly diagnosed cancer were approached in a clinical setting and, after providing informed consent, were screened using the Hospital Anxiety and Depression Scale [17]. Participants with a score greater than 7 on either of the 2 subscales (indicating symptoms of anxiety or depression) were randomly assigned to iCAN-DO or standard care.

**Table 1:** Content and features of the internet-based stepped care in the iCAN-DO application, aimed at individuals with cancer and concurrent symptoms of anxiety and depression.

Step	Description	Who and when?	What?
1	Interactive support guided by a nurse. All communication was in the form of written messages via the U-CARE Portal.	Available for all participants in the intervention group from randomization. Duration 24 months.	The main feature of step 1 was a library containing psychoeducational information and self-care strategies in 16 modules concerning common problems surrounding cancer, such as anxiety, depression, pain, and sleeping issues. Information was delivered in texts, audiovisual presentations, slideshows, and video clips. Some information was directed at all users and some was directed at those with a specific diagnosis, but all contents were visible to all users. In addition to the library, there was a peer-support section and a frequently-asked-questions section. There was also a feature called “Ask an expert,” where users could pose questions to a nurse and read others’ anonymized questions with answers. The nurses were presented in brief, with photos.
2	iCBT^a^ guided by a psychologist. All communication was in the form of written messages via the U-CARE Portal.	Offered only to participants with persistent symptoms of anxiety or depression, or both (>7 on either Hospital Anxiety and Depression Scale subscale) after using step 1. Duration 10 weeks.	Step 2 of the intervention provided a 10-week iCBT treatment program. The treatment contained 15 modules that comprised written texts, audiovisual presentations, and video clips. After completing an introductory module, all participants were free to choose the most relevant modules to work with over the course of 10 weeks. Each module included psychoeducational material, exercises, and assignments and spanned 2 to 4 weeks. Participants were guided by and received weekly feedback from a psychologist who monitored their work and answered any questions they had. A brief presentation of the psychologist, with a photo, was available in iCAN-DO.

^a^iCBT: internet-delivered cognitive behavioral therapy.

### Study Design

This was a qualitative, descriptive study using semistructured interviews and was conducted alongside the AdultCan RCT.

### Informants

Data from the RCT showed that most participants (105/124) used step 1, whereas only a few of those who were offered step 2 in addition to step 1 underwent or were interested in undergoing iCBT (7/82). Thus, through a purposeful selection we strived to include all participants who had used both step 1 and 2, as well as participants who had declined step 2. Since the amount of data necessary to answer the research question was dependent on the quality of data [18], we did not predefine the number of participants at the start of the study, but instead assessed the material for variation and consistencies in statements before ending data collection. We approached 20 individuals, of whom 2 declined to participate and 3 could not be reached. We interviewed 15 informants (Table 2) in 2016-2017. All had had access to iCAN-DO for at least seven months (since step 2 was offered at 1, 4, and 7 months). The informants’ general online activities varied, but all used the internet daily, and 2 informants described themselves as very inexperienced computer users.

**Table 2:** Informants’ characteristics (at time of diagnosis) (N=15).

Characteristics		Values
Age (years), mean (minimum-maximum)		59 (37-69)
**Sex, n (%), and diagnosis**		
	Female	10 (70) (breast cancer)
	Male	5 (30) (1 colorectal cancer, 4 prostate cancer)
**Relationship status, n (%)**		
	Married or partner, living with someone	12 (80)
	Married or partner, living alone	1 (7)
	Widowed	1 (7)
	Single	1 (7)
**Level of education, n (%)**		
	Elementary or middle school	3 (20)
	High school	1 (7)
	University ≤3 years	6 (40)
	University >3 years	5 (30)
**Working situation, n (%)**		
	Working	10 (70)
	Retired	4 (26)
	Early retirement	1 (7)
**General online activity outside iCAN-DO interactive health communication application, n (%)**		
	Daily, no social media	4 (26)
	Daily, active on social media, lurking	7 (47)
	Daily, active on social media, participating	4 (26)
**Activity in step 1 (interactive support), n (%)**		
	Opening material (all sections)^a^ >20 times	6 (40)
	Opening material (some sections) >20 times	7 (47)
	Opening material (some sections) <10 times	2 (13)
**Step 2 (** **internet-delivered cognitive behavioral therapy** **), n (%)**		
	No	9 (60)
	Yes	6 (40)

^a^Library, peer support, frequently asked questions, or ask an expert.

### Procedure

Potential informants were sent a letter regarding the study and informed that the first author would call them within a few days to provide more information and ask them about their willingness to participate. If they did not want to get this phone call, they could email or call the principal investigator for AdultCan (no one used that option). If an informant consented to participate, a time and place for the interview were determined.

### Ethics

All procedures were conducted in accordance with the ethical standards of the institutional and regional research committee and with the 1964 Declaration of Helsinki and its later amendments or comparable ethical standards. All informants were treated with confidentiality and had time to consider their participation after receiving written and verbal information. All provided informed consent before the interviews were conducted. The study was approved by the Swedish Ethics Review Authority (no. 2012/003/9).

### Data Collection

#### Questionnaires and Log Data

We retrieved information on informants’ characteristics and activity in iCAN-DO from self-reported questionnaires and logs in the portal. We retrieved information about online activity beyond iCAN-DO at the time of the interviews.

#### Interviews

The first author (AH) conducted all the interviews. AH is a registered specialist nurse with experience in palliative cancer care. AH has been involved in parts of the development of the stepped care intervention, but has no overall responsibility in the program. AH strived to get as close to the users’ experiences as possible to gain knowledge to facilitate further development of iCAN-DO. The interviews were tape recorded and an interview guide with open-ended questions was used. The first question in the interview guide was “Can you tell me about your experiences of using iCAN-DO?”, then all parts of the system were listed as follow-up questions to ensure coverage, such as “Can you tell me about your experiences of using the library?” Follow-up questions were based on each informant’s responses and used to get deeper descriptions of experiences important to the aim of the study [19]. Specifying and probing questions were used, such as “How did that feel?” “How did you use that?” or “Was there any part that you found helpful/problematic here?,” as well as interpreting questions such as “Do you mean by that...?” One test interview was performed to explore the design and understanding of questions. Each interview was performed in a place chosen by the informant (eg, at home, at their workplace, or at the hospital) and lasted between 45 and 120 minutes. Informants could choose to log in to iCAN-DO during the interview.

### Data Analysis

AH transcribed the interviews verbatim and used inductive content analysis according to Graneheim and Lundman [18] to analyze the manifest content of the interviews. The text was read several times and each interview was divided into meaning units and condensed (reduced while preserving the core). Each condensed meaning unit was then labelled with a code to describe the key message. Codes with similar content were allocated to subcategories and categories (Table 3 shows an example). The analysis was then discussed and assessed within the group of authors several times before reaching the final categories. The main purpose of this validation by the coauthors was to determine that data were labelled and sorted in a way that corresponded not only to the research question, but also to what the informants conveyed in the interviews. The excerpts presented in the paper were translated by AH and Linnea Holmén at Calyptic.

**Table 3:** Steps in the content analysis illustrated by a sample meaning unit.

Meaning unit	Condensed meaning unit	Code	Subcategory	Category
And then I could also show it to my husband, read this, and that was also good because we both got the same information.	I could show it to my husband, read this, that was good because we both got the same information.	My husband and I could get the same information.	iCAN-DO as a source of information for others	A complement to standard care

## Results

### Content Categories

The analysis resulted in 4 categories in the stepped care intervention: (1) gaining knowledge and support but wanting more personalization, (2) a feeling of safety that was needed earlier, (3) own situation, preferences, and timing determine the use of peer support, and (4) a complement to standard care (Table 4). We clarify the context or subject in quotes below in brackets and indicate the 17 subcategories in italic text.

**Table 4:** Categories and subcategories.

Category	Subcategories
Gaining knowledge and support but wanting more personalization	The importance of information and support Confirmation, recognition, and being taken seriously A wish for more specifically tailored contents Positive, supportive contacts with the psychologist Turning down the offer of iCBT^a^ Limitations of iCBT
A feeling of safety that was needed earlier	Reliable and safe to trust Wanted to have access to step 1 earlier Seeking information online started early Information needs vary over time
Own situation, preferences, and timing determine the use of peer support	Not interested in peer support at all, besides lurking Higher presence of health care professionals in the forum Facebook provided a better environment for online peer support
A complement to standard care	Information given at the hospital was insufficient iCAN-DO as a source of information for others Standard care did not offer any support for emotional problems Fit into everyday life

^a^iCBT: internet-delivered cognitive behavioral therapy.

### Gaining Knowledge and Support But Wanting More Personalization

Informants used iCAN-DO to gain understanding of what was going on, both physically and emotionally. The informants described *the importance of information and support* and talked about information gathering and increased knowledge as a way of coping with their new situation. Some of the informants, who had used both steps 1 and 2, discussed how the 2 steps complemented each other.

The first thing you need is information, a foundation knowing that this is correct information that I can trust, this is how it works. Then you need different tools based on that, like for example CBT [cognitive behavioral therapy]. But this thing, the information, it’s the core to understanding the disease. It has to start with that, in order for you to, like, move on.

Female, breast cancer

These modules [step 2] suit me rather well, I have worked with several of them and this first part that is more about self-care [step 1] also suits me quite well....I had pretty big problems with pain, so those parts were important to me; I could get help with that both in iCBT and in the information section [step 1].

Male, colorectal cancer

The content in step 1 was perceived as helpful, calming, and confirmatory. Informants described *confirmation, recognition, and being taken seriously* as factors that could mitigate troublesome thoughts and feelings. Several informants expressed a wish to have access to as much information as possible, even if the information was unpleasant. Symptoms of the disease and side effects of treatment had an impact on daily life and participants considered it a relief to recognize descriptions of their own symptoms or side effects in step 1. This could be achieved through studying information in the library, asking questions, or reading questions from others in the frequently asked questions section. The important thing was to get confirmation that the experiences were well known and real.

Nothing that I read made me sad. It was more like gaining freedom, when reading the answers to questions, for example...to be able to read that this is normal, this is a known side effect, it’s nothing new. And I have been able to use that knowledge many times later on: calm down, this is a common experience.

Female, breast cancer

The possibility of posing questions in step 1 was seen as providing a sense of security and of being taken seriously. The feedback to questions was described as educating and encouraging, although the function would have been more valuable if the nurse who answered questions had access to medical records to provide more specific answers. Furthermore, participants suggested that such a function would be suitable for regular health care.

It’s fantastic, I have gotten such good answers all the time. I must say that “Ask an expert” is the best thing [in step 1] because it feels like someone takes me seriously, someone is giving me a scientifically based answer in words that I can understand. You never have time for those things at the clinic.

Female, breast cancer

I wish that you didn’t have to be put on hold on the phone to make appointments to get advice [in regular health care]. I think that “Ask an expert” should be developed further, as a part of regular health care. You should be able to ask your questions online like this, I think.

Male, prostate cancer

Participants expressed *a wish for more specifically tailored contents* regarding diagnosis, age, the person’s sex, treatment, and symptoms regarding parts of step 1. For example, feeling much younger than other users could create a sense of loneliness. One informant explained that she chose a Facebook forum instead of the forum addressing women with breast cancer in step 1, as she had a need to talk to those with exactly the same diagnosis, prognosis, and treatment.

I can’t identify with a man who has colorectal cancer, for example. We don’t have a lot in common. Not with those who have just breast cancer either. I want to talk to women who have exactly my type of breast cancer [human epidermal growth factor receptor 2-positive]. We discuss things based on our diagnosis and situation, because it is different, like survival and side effects of medicines and how we feel ahead of the surgery, things like that....

Female, breast cancer

The informants who underwent the iCBT program (step 2) in addition to step 1 experienced *positive, supportive contacts with the psychologist* and materials that were useful for managing symptoms. The feedback was described as encouraging and genuine and was also a trigger to log in and participate further. Undergoing iCBT was described as demanding, but worth putting effort into. Informants felt that they got help and that, while symptoms such as pain, anxiety, or insomnia would still be present, iCBT gave them tools to manage the situation.

My anxiety was relieved, absolutely; it felt like...when working with the [cognitive behavioral therapy] program...going from constant anxiety, I could use what I had learned. I’d think, that’s right, now I’ll do this...and then the anxiety faded. Then it might come back, but then I can use what I have learned again. I now have the tools. I still think that way.

Female, breast cancer

A feeling of sufficient support from relatives and friends was a reason for *turning down the offer of iCBT* provided in step 2. A fear of “making it worse” by focusing on the negative feelings was also mentioned as a reason, as were past experiences affecting confidence in the method or already having a professional contact. Among the informants who participated in step 2, experiences of its helpfulness varied and several *limitations of iCBT* were described. A common reflection was that the time limit set for therapy (10 weeks) was too short in an already strained situation. Choosing among modules was sometimes difficult and a feeling of “needing them all” was experienced as stressful. The division into modules could also be perceived as difficult, as all the symptoms interacted. Symptoms and needs were described as dynamic and changing during the period of illness. Informants understood the purpose of the set time frame and modules, but still found it hard to deal with. One informant described choosing to work with self-help apps instead, because then time was not a problem.

When I realized that I was supposed to finish all that in those weeks I felt, “no”...because that’s a very intense schedule, oh I felt such pressure. I just felt I wouldn’t manage, so I didn’t finish it. Since I got my diagnosis, my sensitivity to stress has been, well...I can’t take any stress, zero.

Female, breast cancer

Some experienced the online delivery of the treatment and interaction with the psychologist by written messages as positive because they felt that they could form the level of relationship they needed. The use of written communication was described as a kind of processing in itself and as a way to get a grip on one’s own thoughts. One informant stated that cognitive behavioral therapy in a real-life context would not be as appealing. Communicating with the psychologist in writing could also be experienced as limiting and seen as an obstacle by some, who felt that the psychologist was absent and uncaring. A video meeting at some point during treatment was one suggested facilitator. Informants further suggested that the contacts with the psychologist should be visible in medical records, to facilitate continued work with a psychologist in a real-life context.

It was almost fascinating that it worked so well, but also a bit strange that someone that you never meet knows so much about your life...it’s strange and astounding...it was a new kind of relationship that I have never experienced before.

Female, breast cancer

It wasn’t at all the relationship I wanted; this was faceless. I felt like the line of text that I got from the psychologist had probably been sent to 10 other people that day. Like...it wasn’t about me.

Female, breast cancer

### A Feeling of Safety That Was Needed Earlier

The information and self-care advice provided in step 1 was perceived as *reliable and safe to trust*. Informants highlighted it as positive that they could be sure there were no hidden agendas or profits involved, as that was something perceived to make other online information difficult to evaluate. Evaluating information on the internet could be strenuous and time consuming. Informants described looking for confirmation in step 1, using it to judge whether something they had been told or read elsewhere was accurate. Another aspect of security was that of knowing that there was easily available and reliable information in step 1, in case it was needed in the future, even if it was not actively used at the time. Having access to information was experienced as a privilege.

Because there is such a wealth of information on the internet, I think it has been good to have information from an official page, so to speak; it felt good. It toned down the whole thing a bit. There are horrible stories on the internet about the side effects of the treatment. On various forums on the internet, medical knowledge is questioned.... So, therefore, I stopped searching the internet. I haven’t used the internet all that much; it's mostly the portal I’ve used.

Female, breast cancer

Many of the informants described that they would have *wanted to have access to step 1 earlier*, preferably when informed of the diagnosis. That occasion had been followed by large information needs, and informants looked for information that could give them a sense of control. *Seeking information online started early* in close proximity to gaining knowledge about the diagnosis during the medical investigation, or even before. Many had already searched for information online when gaining access to step 1 and some had bad experiences from such online information. The informants who reported that they used online forums for support described that they had already found forums elsewhere when they got access to the portal. Informants also described how their *information needs would vary over time*, and it was seen as important to have access over a period of time, as the material could be used at different times during the illness, depending on individual information needs.

You should get access at once, at the point when your world falls apart, because that’s the time when you need the answers. To me, it didn’t matter that the doctor said that I would most likely get well, I still needed to read the facts myself and I needed to do that right away.

Female, breast cancer

Sometimes you may not get full insight into your own situation at once, it takes time...and then you should have access to this when the right time comes...it takes time, it comes later...and I am the kind of person who...I don’t want to bother others, some can argue for themselves but no, I can’t, since I’m not like that. So for me, it’s actually better that it is available like this.

Male, prostate cancer

### Own Situation, Preferences, and Timing Determine the Use of Peer Support

When asked about the section containing peer support, most of the informants said they were *not interested in peer support at all, besides lurking*. This could have several different causes, such as preferring professional support or peer support face-to-face, not being the “social type,” feeling that peer support was just opinions often not based on facts, or fearing that discussions would degenerate into being nonsupportive. Others said that they might have used peer support more if they did not have family and friends to talk to. Some of those describing themselves as not interested declared that they sometimes read forums, both in step 1 and elsewhere online, but that they would never write something themselves. Some would consider active participation if health professionals were more involved, and a *higher presence of health care professionals in the forum* in step 1 was suggested. Some informants found peer support in general to be highly important and helpful but expressed that *Facebook provided a better environment for online peer support*. The informants had all already found Facebook groups when they got access to step 1 and described both positive and negative experiences from this. They also stated that the forum in step 1 could not compete with the Web environment, easy access, and high specificity and activity in Facebook groups. However, some informants described a lack of moderators and a nonsupportive environment in Facebook groups.

Well, I didn’t look in here so often. I think it’s because I have a lot of people around me to talk to. If I was alone it would have been different, because you need to have something...but since I can choose, I’d rather sit down and talk to someone.

Male, prostate cancer

### A Complement to Standard Care

Informants described step 1 as a complement to standard health care in several ways. It was useful in the cases when *information given at the hospital was insufficient*, for example, that it was brief or difficult to understand. Being in a state of shock when getting the news of the diagnosis also made the information at the hospital hard to remember, and there was a need for repetition in step 1. Informants were relieved to see that the information given in the hospital was consistent with the information in step 1. The information could also be used to prepare for hospital visits. All informants stated that factual information and self-care advice were highly appropriate to provide in a Web-based form, but some stressed that they preferred emotional support in a real-life context.

You should have a system like this with the opportunity to get in touch with a person in health care. It doesn’t have to be a physician, but a person connected to the clinic, because you feel a lot of anxiety in between medical examinations and such.

Male, prostate cancer

I booked an extra meeting with the physician because I was so scared about the radiation, and, sure, I got answers from the physician, but then I could get it confirmed through the material here, and the information here was consistent with what he said. That fact made me feel safer.

Female, breast cancer

Informants further described both step 1 and step 2 in *iCAN-DO as a source of information for others*. Relatives were described as being left out and it could be burdensome to manage their worries and questions. Informants described how relatives could have trouble trusting information from informants and that it was an advantage to be able to read the same information together.

Well, I let her read all this. She thinks it’s good, and good that I have something to do as well. We also discussed things with the material as a starting point; I think it’s good. Some of the material describes...well, relations and such. This is something that we must handle, between us, with those close to us.

Male, colorectal cancer

I felt a confirmation, a sense of security regarding the disease, and I felt that I could trust what was said. Since it was published here in writing, then I could also show it to my husband, “read this,” and that was also good. We both got the same information.

Female, breast cancer

When talking about both steps in iCAN-DO, several informants mentioned that *standard care did not offer any support for emotional problems*, and some said that even if they thought that they could get support from a counselor, they did not know whether it would be helpful, or did not want to burden health care services. The fact that iCAN-DO included emotional, practical, and bodily issues was noted by some informants who described standard health care as separating the body from the emotions, even if emotions emanated from a bodily problem.

No, this doesn’t exist in regular health care and sometimes when I was in the portal I felt at least I am not completely alone, there are others whose situations are like mine. I also saw those who felt even worse than I did...I don’t see it like...well, this was not a complement, this was the only, the one support that I got.

Female, breast cancer

It was important to be able to read this [in the step 1 library] at my own pace, but I also wanted to pose my questions to a real person. When I got the relapse, I told the physician that it felt quite stressful and the answer I got was, well, there are counselors...and I think that is so...well, it’s the worst thing I know! You can’t divide yourself up like that...a lot of worry is connected to the body.

Female, breast cancer

Further, it was difficult to fit standard health care into everyday life. Informants stated that life did not stop or slow down when they got ill: their work had to be done, families had to be taken care of, and so on. Working informants experienced a lot of stress, and they appreciated being able to use the stepped care intervention when it *fit into everyday life*. Both working informants and those who were retired or on sick leave described difficulties in getting in touch with regular health care services during office hours and having limited time to get questions answered by regular health care providers. iCAN-DO was a possibility to get support and answers close to when a need for it occurred and when the informants had time. Informants also talked about features in relation to standard health care and some suggested possible implementations.

You are not bound to a set time—you can do it anytime you wish in the evening, for example—that’s the biggest gain...if you have time in the middle of the night, you can sit down then.

Male, colorectal cancer

It should be easy, because you can’t call the clinic all the time, and they may not be able to answer when you call...it's hard to get in touch, so it was here in the library I found the answers and that was good.

Female, breast cancer

## Discussion

### Principal Findings

Participants experienced iCAN-DO as a useful and reliable source of information and support and used it as a complement to standard care. They described the need for information as being large and looked upon the gathering of information almost as a survival strategy when cancer was newly diagnosed. To be able to understand and handle the entire situation, informants expressed a need for increased knowledge, a result that corresponds well with the theoretical basis of the intervention, where psychoeducation is a prominent feature. They suggested that knowledge was the foundation for continued processing, implying that the stepped care model in iCAN-DO would be suitable. In addition, informants often talked about the stepped care intervention in relation to standard health care, where they often felt that they were not seen as whole individuals, but rather as a set of separate symptoms.

The overall picture was that standard health care did not seem to suit their needs, leaving them with a lot of unanswered questions and a lack of emotional support focused on their cancer type and its treatment. The high, often unmet needs for information and psychosocial support among individuals with cancer is well known [20]. Before gaining access to step 1, informants sought answers on the internet, an action previously described as the first choice in the absence of contact with health care professionals [21,22]. Here iCAN-DO seemed to fill a void, providing easy access to information that could be trusted. It is essentially positive that individuals look for information that can facilitate the handling of disease, but the quality of this information could be better ensured if integrated as a natural part of routine care, which informants also suggested. Most informants described lurking (nonactive participation) in forums, both in iCAN-DO and elsewhere online, and this is by far the most common behavior in internet forums, including those regarding behavior change and health [23]. By reading about other people’s experiences, the lurker may learn and benefit on a personal level, but still a forum needs active participants to stay alive and healthy. Informants in this study suggested that they would be encouraged to participate by more active participation from health care providers, and this is in line with a literature review [24] suggesting that moderators should create opportunities for delurking by providing new forum members with supportive, encouraging information, as well as highlighting the value of contribution. Such attitudes could be further developed within iCAN-DO forums.

The need for information and support is the highest at the time of diagnosis [20], and the timing of the intervention was also highlighted by most informants, who would have wanted access to step 1 immediately at the time of diagnosis. Information seeking was sometimes described as having started in the early investigation phase. During the development of iCAN-DO, the clinical staff and those with lived experience of cancer agreed that it was best not to approach presumptive study participants immediately at the time of diagnosis. This was decided because shock and denial might be present [2] and the question posed would be regarding participating in the AdultCan RCT, with no promises of additional psychosocial support. Also, the time of diagnosis and start of treatment imply an intensive period with diagnostic procedures, loads of information about the treatment, and possible inclusion in clinical trials. Thus, we regarded the time of diagnosis as inappropriate for inclusion in AdultCan. However, the findings of this study suggest that when implementing evaluated internet-based support in routine clinical care, the time of diagnosis could be a suitable occasion for introduction.

iCBT was fitting and helpful for some, but not all. For some, the ability to handle symptoms seemed to increase, while others seemed to require the face-to-face presence and guidance of a psychologist. The burden and strain of the illness and treatment itself seemed to negatively affect the possibilities of engaging in a fixed, time-limited therapy like iCBT. Adjustments to a set time frame during iCBT have previously been described as inefficient [25], suggesting that iCBT treatment should be agreed upon and adapted to the individual at the initial stage.

Besides the specific burden of illness and treatment, participants’ perseverance may be affected by the fact that they did not actively seek support. Individuals undergoing iCBT both in trials and in clinical settings are often recruited via the internet and by self-referral [26,27]. Since our sample consisted of individuals with a recent diagnosis of cancer, recruited in a clinical setting, the program would likely have gained from being more adaptable to the individual situation. A previous evaluation of a similar iCBT intervention for individuals who had myocardial infarction [28] suggested that individuals’ technical skills, personal preferences, and life context must be considered, and other results suggested that individuals with comorbidity benefit more from a tailored iCBT program than from one-size-fits-all protocols [29]. Another study on the feasibility of iCBT for individuals with a new diagnosis of cancer [30] reported that some of the 13 informants interviewed found it difficult to complete the program. Further, participants in that study appreciated the flexibility and private nature of the iCBT program that was provided but suggested that they wanted supplementary content about the side effects of treatment. This might further support the stepped care approach in iCAN-DO, which offered information and self-care strategies for side effects in step 1, in addition to iCBT. Again, support for this group may be more relevant if aiming to address the totality of their situation following the cancer rather than focusing on individual symptoms.

Informants appreciated the possibility to ask an expert in step 1 but stated that it would have been even more useful if the nurse had access to medical records, and they also wanted the psychologist’s treatment to be visible in the medical records. This suggests that the intervention would have gained from being integrated into standard care. There were thoughts of a system tethered to electronic health records in the development stage, but unfortunately the lack of interoperability and lengthy legal processes would most likely have delayed the project for years. Thus, further development and cooperation between stakeholders is much needed. These needs and possible solutions have recently been described in depth by Signorelli et al [31], who also suggest that the integration of electronic health records can increase personalization.

Further, the informants used iCAN-DO to inform next of kin, who were described as being left out. Earlier investigations have shown that next of kin are at risk of developing symptoms of anxiety or depression [32] and, as eHealth resources have shown the ability to decrease both perceived burden and negative mood symptoms among next of kin [33], we plan to include information and support specifically directed at them in our forthcoming projects.

Despite positive experiences of iCAN-DO, the common denominator was the desire for individual tailoring to one’s own situation and needs. Informants suggested that the intervention should target them more specifically, not only on the basis of having cancer and concurrent symptoms of anxiety or depression. The results suggest that, for example, diagnosis, type of treatment, symptoms, age, and the person’s sex could be taken in consideration when tailoring the support. We based the design of iCAN-DO on an evidence-based triad [15] of clinical expertise, best scientific evidence, and patient involvement. In retrospect, we could have aimed at a more heterogeneous group of individuals with lived experience of cancer. As Bandura described, attempting to tailor health communications is no guarantee of a positive outcome, since the benefits depend on the value of the tailored factors [34]. Future interventions could probably gain from being integrated into standard care, as it may be easier to achieve more relevant personalization there than in a separate research context. Further, some previous studies have used tracking of symptoms as a means to tailor the content by, for example, suggesting material to the user [35-37]. In addition to these possible adjustments, the delivery mode and design of the program and its included features must be considered in regard to the entire user experience. We have analyzed these aspects of iCAN-DO separately (HI, unpublished data, 2020).

### Methodological Considerations

The purposeful sample strategy aiming to include participants who used both step 1 and step 2 and participants who had declined step 2 resulted in a predominance of female informants with breast cancer. This reflects the total sample in the AdultCan RCT, in which this group was the largest, followed by those with a diagnosis of prostate or colorectal cancer. Informants still varied in other aspects such as general online activity, level of education, and age. The informants in this study had access to a specified internet-based stepped care intervention and may therefore be considered a “specific sample” according to Malterud et al [38]. Their description of sample sizes in qualitative studies suggested that a smaller sample size is sufficient when the anticipated specificity in informants’ experiences is high. Thus, when we had interviewed 15 informants, we assessed the material and, as statements seemed to be both varied and consistent, we terminated data collection. However, we interviewed only 2 informants with a low activity level in iCAN-DO, since they had difficulties discussing the topics covered in the interview guide, which might have caused them some discomfort. The purpose of the interview guide was to provide dependability and stability over time and under varying conditions [19], thus strengthening credibility. The low-activity users could be interviewed separately, using a different research question, to further explore possible customizations of the program.

Regarding transferability, both the content of iCAN-DO and the sample of informants were specific, but the needs for information and support may be similar in individuals in other diagnostic groups. Also, we have strived to describe iCAN-DO and the surrounding conditions in a way that allows those interested to determine whether results are usable in another context, such as when developing an internet-based support program. Issues of confirmability must be taken into consideration in that informants might have been affected by the fact that someone working within the project conducted the interviews. Even though the interviewer assured informants that she was focused on program development, they might have expressed their opinions in more positive terms than they would have if someone completely independent had conducted the interviews. However, an independent interviewer might have affected the quality of the dialogue due to a lack of knowledge about the contents and features of iCAN-DO. Further, regarding confirmability, the analysis described in the Methods section was conducted mainly by the first author but then assessed and discussed within the group of coauthors at several occasions during the process. The group of authors consisted of individuals with expertise in nursing, psychology, and physiotherapy, although 1 of the coauthors contributing to analysis had nothing to do with iCAN-DO.

### Conclusion

iCAN-DO was experienced as a safe and reliable source of information and support, but informants highlighted the importance of individualized information, support, and delivery in both step 1 and step 2. Further, internet-based support should be offered close to the time of diagnosis and would gain from being integrated into standard care. Future trials should focus on how to personalize and integrate internet-based information and support into clinical everyday life and, meanwhile, the clinic should assist patients by guiding them to evidence-based, reliable sources on the internet.

## Abbreviations

eHealth: electronic health
iCBT: internet-delivered cognitive behavioral therapy
IHCA: interactive health communication application
RCT: randomized controlled trial
